# Medicine

**Published:** 1904-12

**Authors:** J. Michell Clarke


### Progress of the Medical Sciences.

### MEDICINE.

**Lumbar Puncture and Cyto-diagnosis of Cerebro-Spinal Fluid.** —Donath's[1] experiences of lumbar puncture in 98 cases of meningitis, brain abscess and tumour correspond with those of other authors. He points out that only so much fluid should

[1] *Wien. med. Wchnschr*, 1903, liii. 49.

be withdrawn as will flow freely, and that it should never be aspirated. The little operation should, of course, be done under antiseptic precautions. The normal fluid should be clear as crystal, and the slightest turbidity is pathological. As a therapeutic measure, he found lumbar puncture of value in meningitis and brain abscess, and in relieving the gastric crises and lightning pains of tabes. In 19 cases of epilepsy, although of course no cure was effected, the fits were fewer and sleep and the general condition improved.

Rhodes[1] also claims for lumbar puncture relief of pain, and of coma or loss of consciousness due to increased intra-cranial pressure; in one case of basal meningitis it aided recovery. Both authors emphasise its usefulness in diagnosis in difficult cases. This point will be dealt with immediately.

Other authors attribute both alleviation of symptoms and more lasting improvement to lumbar puncture in many cases of nervous disease.

Babinski[2] states that lumbar puncture relieves, or even cures, the symptoms of Menière's disease, and may favourably influence deafness and tinnitus aurium; but its field of usefulness in the latter affection is a limited one. It is most likely to be effectual in cases of pure labyrinthine disease.

Legrain and Guiard[3] report a case in which very severe headaches in the course of Bright's disease were relieved by lumbar puncture, which was done 7 times.

**Cyto-diagnosis.**—Much attention has lately been directed to the character of the cells present in the cerebro-spinal fluid in various diseases as an aid to diagnosis. The method of procedure is to centrifugalise for 15 minutes the fluid obtained by lumbar puncture, to make a cover-glass film from the deposit, dry it at 37° C., fix it with alcohol and ether, and stain with methylene blue or hæmatoxylin and eosin (or Jenner's stain may be used, which does not require fixation of the film). The normal cerebro-spinal fluid is almost entirely free from cells; those present from pathological processes may be red blood cells, polymorphonuclear leucocytes, which may be neutrophil, eosinophil or basophil, and lymphocytes in varying proportions. In cerebro-spinal meningitis there is a marked leucocytosis (polymorphonuclear cells),[4] in tuberculous meningitis a lymphocytosis, and in all chronic diseases of the central nervous system with affection of the meninges a pronounced lymphocytosis.

In cases of difficult diagnosis Chauffard and Boidin,[5] whose paper deals with 140 cases and 223 separate punctures, found the procedure of special value; for instance, in some cases

1 *Brit. M. J.*, 1903, ii. 73.

2 *Ann. d. Mal. de l'Oreille et du Larynx*, 1904, xxx. 101; abstract in *Neurol. Centralbl.*, 1904, xxiii. 863.

3 *Progrès méd.*, 1903, 3e. sér. xviii. 281.

4 Demange, *Inaug. Diss.*, Paris, quoted in *Schmidt's Jahrb.*, 1904, cclxxxiii. 239.

5 *Gaz. d. Hôp.*, 1904, lxxvii. 725.

of acute infectious fevers, in spite of pronounced meningeal symptoms, meningitis could be excluded by this means.

To take now the diseases in which there is decided lymphocytosis.

In **Tabes Dorsalis** a more or less pronounced lymphocytosis seems to be the rule. A number of cases were brought forward by various observers at the Société de Neurologie of Paris,[1] and with others that I have collected from various sources form a total of 98 cases; lymphocytosis was marked in 85, absent in 7, and slight in 4: these last and one of the negative results were cases of very old standing. Drs. Marie and Crouzon in 20 cases were not, however, able to trace any connection between the degree of lymphocytosis and the severity of the other symptoms.

In **General Paralysis** the results are equally striking. Of 28 cases reported by different writers 25 showed lymphocytosis.

Remembering the undoubted connection between these diseases and syphilis, the question suggests itself as to whether the lymphocytosis is due to the latter. In this connection Ravaut[2] states that in many cases of syphilis lymphocytosis of the cerebro-spinal fluid is present during the whole course of the disease, and considers that this shows a reaction of the meninges at a time when the cerebro-spinal lesions of syphilis are absent. Other observers have also reported a pronounced lymphocytosis in various syphilitic affections of the nervous system. Niedner and Mamlock[3] found in 6 hemiplegics, lymphocytosis in 4, in whom the hemiplegia was due to syphilis. In these 4 cases syphilis, and not hemiplegia, must be regarded as the cause of the lymphocytosis; but they consider that this does not hold good for tabes, for 3 of their cases gave a positive result in the absence of any evidence of syphilis. Babinski[4] states that lymphocytosis is not pathognomonic of a syphilitic meningitis in tabes dorsalis.

Niedner and Mamlock[5] conclude that lymphocytosis results from severe and long-lasting irritation of the central nervous system, such irritants on the one hand being intoxications, syphilis, uræmia, tetanus, &c., and on the other of a mechanical nature, tumours, &c. They do not look upon meningeal irritation as the most essential factor in lymphocytosis, and conclude with regard to tabes that seeing that lymphocytosis is frequent in syphilis, and may be absent in tabes, a positive or negative result of the examination of the cerebro-spinal fluid would not settle the diagnosis of a doubtful case.

**Tuberculous Meningitis.** — Chauffard and Boidin[6] in 13 cases obtained a positive result in all, and in 9 tubercle bacilli

[1] *Neurol. Centralbl.*, 1904, xxiii. 873 *et seq.*
[2] *Lyon méd.*, 1903; abstract in *Jahresb. d. Neurol. u. Psychiat.*, 1904, i. 532.
[3] *Ztschr. f. klin. Med.*, 1904, liv. heft 1 u. 2; abstract in *Neurol. Centralbl.*, 1904, xxiii. 863.
[4] *Neurol. Centralbl.*, 1904, xxiii. 874. [5] *Loc. cit.* [6] *Loc. cit.*

were also found in the cerebro-spinal fluid. F. and A. Torday[1] found in 10 cases pronounced lymphocytosis in all; there was also a slighter increase of polynuclear cells. In 5 cases they discovered tubercle bacilli in the fluid. Variot and Percheron[2] emphasise the value of this lymphocytosis in the early diagnosis of the disease; but F. and A. Torday and other observers say that taken alone it may lead to error, and must be considered in connection with the other symptoms.

Heischhorn,[3] as the result of experiment, has found that guinea-pigs, injected with the cerebro-spinal fluid of a person suffering from tuberculous meningitis, always become tuberculous, but when injected with this fluid from cases of tuberculosis of other organs are not infected.

Orglmeister,[4] from observation of 15 cases of tuberculous meningitis, says that clotting of the cerebro-spinal fluid, or the presence of flakes of fibrin and of leucocytes in it, are not sufficient without the demonstration of tubercle bacilli for the diagnosis of the disease from the characters of the fluid alone.

In a case in which there were numerous tuberculous foci in the cerebellum, one of which was situated on the surface and gave rise to a local meningitis, Nobecourt and Voisin[5] found a lymphocytosis and tubercle bacilli in the cerebro-spinal fluid.

Pernet[6] reports 86 cases of meningitis. Most were tuberculous in nature, only 7 being acute and non-tuberculous. Lumbar puncture was done 53 times, and gave a lymphocytosis for most tuberculous, and a leucocytosis for all the acute cases. In these acute cases the cause of the meningitis was in some Weichselbaum's meningococcus, in others the pneumococcus and Pfeiffer's bacillus.

Other diseases in which lymphocytosis was found on examination of the cerebro-spinal fluid are herpes zoster (to a very marked degree), parotitis, acute anterior poliomyelitis (one case), and urticaria. In disseminate sclerosis J. Fraenkel[7] reports a pronounced **leucocytosis** in 6 out of 7 cases. Most observers say there is no lymphocytosis in tumours of the brain, but it has been found once or twice. A negative result has been obtained in epilepsy, softening of brain, hysterical nervous affections, aural vertigo, poliomyelitis, syringomyelia, old-standing hemiplegia, multiple neuritis, and functional neuroses.

Accompanying lymphocytosis, but independent of it in origin, Guillain and Paraut[8] have found albumin in the cerebro-spinal fluid in 36 patients suffering from general

1 Abstract in *Jahresb. d. Neurol. u. Psychiat.*, 1904, i. 532.
2 *Ibid.*, p. 531. 3 *Thèse de Paris*, 1903; *Ibid.*, p. 531.
4 *Deutsches Arch. f. klin. Med.*, Bd. lxxvi. 142.
5 *Rev. mens. d. Mal. de l'Enf.*, 1903, xxi.; abstract in *Neurol. Centralbl.*, 1904, xxiii. 31.
6 *Thèse de Paris;* abstract in *Jahresb. d. Neurol. u. Psychiat.*, 1904, i. 495.
7 *Med. Rec.*, 1904, lxv. 125. 8 *Rev. de Neurol.*, 1903, 406.

paralysis, disseminate sclerosis, chronic alcoholism, dementia præcox, epilepsy, and melancholia. In the general paralytics there was an increase in the globulins and a decrease in the serum albumin.

With regard to the **colour of the fluid** withdrawn by lumbar puncture, Professor Bard[1] states that centrifugalised cerebro-spinal fluid containing blood shows, if the hemorrhage has just occurred, a clear, colourless fluid above the deposit; if the hemorrhage is older this is rosy red, and the presence of hæmoglobin can be demonstrated; if several weeks have elapsed the fluid is a clear yellow. These appearances he attributes to a hæmolytic action by the pathological cerebro-spinal fluid on the red blood corpuscles.

Mathieu[2] also attaches diagnostic significance to the colour of the cerebro-spinal fluid in hemorrhages. If the fluid is brown or greenish brown, a pathological process is present; if it is mixed with blood, this may be either due to the puncture or to hemorrhage from the brain or cord. If after centrifugalisation the fluid is colourless, the blood is of artificial origin (puncture); if the fluid is, however, brown or greenish brown it is pathological.

Duprè and Sébilleau[3] have been able in a case of cerebral hemorrhage to diagnose hemorrhage into the ventricle from the presence of red blood cells in the cerebro-spinal fluid. In a similar case Sabrazès and Muratet[4] found in the centrifugalised fluid 54 per cent. large mononuclear cells, many of which contained hæmatin.

Mongeur[5] has found very frequently a slight increase of the **reflexes after lumbar puncture** in various diseases of the nervous system.

Although the little procedure of lumbar puncture is in the great majority of cases simple and harmless enough occasionally **disagreeable symptoms and even death** have been known to follow it.

The commonest symptoms are pains in the head, vomiting, or nausea, and faintness. These symptoms are mostly temporary and soon pass off; they are attributed by Donath[6] to hyperæmia of the meninges and cortex cerebri, due to a rapid fall of intracranial pressure.

Ballet and Delherm[7] say that such symptoms are most often met with where the cerebro-spinal fluid is normal. They occur in only a very small proportion of the cases examined.

Masing[8] reports a case in which death followed 15 hours after a lumbar puncture had been done, and was due to a large hemorrhage in a gliosarcoma of the temporal lobe; he attributes

[1] *Semaine méd.*, 1903, No. 41; abstract in *Neurol. Centralbl.*, 1904, xxiii. 860.
[2] Abstract in *Jahresb. d. Neurol. u. Psychiat.*, 1904, i. 497.
[3] *Neurol. Centralbl.*, 1904, xxiii. 874. [4] *Compt. rend. Soc. de Biol.*, lv. 1435.
[5] *Ibid.*, 1561; abstract in *Jahresb. d. Neurol. u. Psychiat.*, 1904, i. 387.
[6] *Loc. cit.* [7] *Soc. de Neurol. de Paris*, *loc. cit.*
[8] *St. Petersb. med. Wchnschr.*, 1904, xxix. 1; abstract in *Schmidt's Jahrb.*, 1904, cclxxxiii. 58.

the hemorrhage to a rapid lowering of the intracranial pressure. He is of opinion that lumbar puncture is worthless and dangerous in brain tumours.

J. Fraenkel's[1] view is—he had a case of cerebellar tumour in which collapse occurred after lumbar puncture—that it is in cases of disease in the posterior fossa, and especially of the cerebellum, that there is danger in lumbar puncture, as these are the cases in which death has followed it. He therefore advises that it should not be done in such cases.

J. MICHELL CLARKE.

